# Mammography Compliance for Arizona and New Mexico Hispanic and American Indian Women 2016–2018

**DOI:** 10.3390/ijerph21010019

**Published:** 2023-12-22

**Authors:** Carol M. Seanez, Tomas Nuño, Francine C. Gachupin, Robin B. Harris

**Affiliations:** 1 Department of Epidemiology and Biostatistics, Mel and Enid Zuckerman College of Public Health, University of Arizona, 1295 N Martin Ave., Tucson, AZ 85724, USA; tnuno@arizona.edu (T.N.); rbharris@arizona.edu (R.B.H.); 2 Department of Family and Community Medicine, College of Medicine, University of Arizona, 1601 N Tucson Blvd., Ste 32, Tucson, AZ 85716, USA; fcgachupin@arizona.edu

**Keywords:** screening, healthcare access, cancer disparities

## Abstract

Hispanic and American Indian (AI) women experience lower breast cancer incidence than non-Hispanic White (NHW) women, but later-stage diagnoses and lower survival rates, suggesting issues with screening and healthcare access. Between 1999–2015, NHW breast cancer incidence decreased by 10% but increased by 8% for AI women. This study used 2016 and 2018 Behavioral Risk Factor Surveillance System data for Arizona and New Mexico to explore mammography screening. Analyses included Hispanic, AI, and NHW women aged ≥40 years (*n* = 12,830) to calculate age-specific compliance by race/ethnicity, logistic regression, and adjusted and sample-weighted evaluated associations between compliance and socio-economic covariates. In total, 75.1% of Hispanic women aged 50–74 reported mammography in the past two years (United States Preventive Services Task Force compliant) compared to 73.9% of NHW and 71.0% of AI women. Women who reported doctor visits in the past 12 months were likelier to comply than those without (AOR = 4.2 for Hispanic, 2.9 for AI, and 3.2 for NHW women). Reporting access to a personal doctor was related to compliance, except for AI women. While screening compliance was over 74%, visiting a healthcare provider in the past 12 months was essential. AI women reported issues that suggest unique challenges when deciding on mammography.

## 1. Introduction

In the United States (U.S.), one in eight women will be diagnosed with invasive breast cancer, the most common cancer that impacts women globally when skin cancer is excluded [1,2,3]. The incidence and mortality of breast cancer are not the same for different racial and ethnic groups. American Indian (AI) and Hispanic women have lower breast cancer incidence than non-Hispanic White (NHW) but experience more late-stage diagnoses and larger tumors [1,4]. Overall, 61.6% of Hispanic and 61.7% of AI women have a localized tumor compared to 68.4% of NHW women [1,5,6,7,8]. There are few studies that have focused on the association between USPSTF guideline compliance and variables associated with health utilization and sociodemographic characteristics for American Indian and Hispanic women living in the Southwest U.S.

Although many studies indicate that incidence is lower for AI women than NHW, a study from 2019 found that breast cancer incidence decreased by 10% for NHW but increased by 8% for AI between 1999 and 2015 [9]. Differences in breast cancer incidence amongst Hispanic and AI women compared to NHW might be due to differences in reproductive patterns, such as first childbirth at a younger age, greater number of children, and longer duration of breastfeeding. One study noted that each full-term pregnancy was associated with a 12% reduction in post-menopausal breast cancer risk [2].

Breast cancer screening has significantly reduced breast cancer mortality, so breast cancer screening rates and compliance are important factors to assess [2,4,10]. Higher cancer-related mortality rates amongst racial and ethnic groups compared to NHW are attributed to cancer screening disparities [10].

Various national organizations established guidelines for breast cancer screening [2]. Based on breast cancer simulation models, the United States Preventive Services Task Force (USPSTF) provided updated screening guidelines in 2009 for asymptomatic average-risk women [11]. However, this set of recommendations conflicted with other organizations like the American Cancer Society (ACS), which recommended that screening begin at age 40 [11]. The ACS updated its recommendations in 2015 to include (1) yearly mammography from age 45 to 54 and (2) mammography every two years for those 55 and above who were in good health and had at least a life expectancy of 10 years or more [12]. The American College of Radiology (ACR) updated its recommendations in 2017 to state that women aged 40 and above should be tested yearly for mammography and should stop based on a woman’s health status and not their age [13]. The Center for Disease Control and Prevention (CDC) adopted the USPSTF recommendations, as evidenced by their use in CDC-funded programs and website [13,14,15]. Table 1 summarizes the different guidelines recommended by the three organizations.

The utilization of breast cancer screening protocols is considered to have reduced breast cancer mortality by 15–40% because it can help detect cancer before symptoms develop and decrease the progression to metastasis [10]. This is important because increasing tumor size and grade are inversely correlated with survival rate [17,18]. In 1990, Congress passed the Breast and Cervical Cancer Mortality Prevention Act, prompting the CDC to create the National Breast and Cervical Cancer Early Detection Program (NBCCEDP) to address the issue of screening underutilization in underserved areas [19]. AI women have historically utilized the NBCCEDP at a higher proportion (33%, 2011–2012) than all other races and ethnicities (8.7–12.2%) [9]. Despite evidence of AI breast cancer screening increasing between 2008–2015, the screening rate is still below the Healthy People 2020 target [9]. Other laws passed that aim to increase breast cancer screening and treatment include the Breast and Cervical Cancer Prevention and Treatment Act of 2000 and the Native American Breast and Cervical Cancer Treatment Technical Amendment Act of 2001 [19]. In 2010, the Affordable Care Act (ACA) was signed into law, expanding healthcare coverage in the U.S. [20]. A study showed that after the ACA passed, eligibility for the NBCCEDP decreased. However, the program still serves less than the number of women who qualify [20].

Previous studies have found that education level, socioeconomic status, current health insurance, and having a regular physician impact mammography screening rates [18,21]. Duggan noted that lack of education, health insurance, a primary physician, and low income were all associated with lower breast cancer screening [18]. Lack of health insurance was the strongest indicator of breast cancer screening utilization [22]. AI women who were not eligible for Indian Health Service (IHS) services were more than three times as likely to be diagnosed with late-stage breast cancer than NHW [23]. Another observed barrier to mammography utilization was rural residency. Two studies reported that, overall, AI and Hispanic women in Arizona had similar cancer screening rates as NHW, but women living in rural counties had lower breast screening rates than those living in urban settings [7,24].

In Arizona and New Mexico, Hispanics comprise the largest minority group (32.3% and 50.1%, respectively), with Mexican-origin individuals encompassing the largest subgroup [7,25]. Also, there are 45 federally recognized tribes between the two states, with AI making up 5.3% of the Arizona population and 11.2% of New Mexico [25]. In this study, we utilize survey data from the CDC Behavioral Risk Factor Surveillance System (BRFSS) to determine the current mammography screening experience of AI, Hispanic, and NHW women and identify sociodemographic and healthcare experiences that are associated with compliance with USPSTF guidelines.

## 2. Materials and Methods

### 2.1. Methods

#### 2.1.1. BRFSS

The BRFSS is a national telephone survey that was started in 1984 by the CDC to collect data on behaviors that would be useful for planning, initiating, supporting, and evaluating health promotion and disease prevention programs in the U.S. [26]. It is now the largest continuously conducted health survey system in the world that collects health-related risk behaviors through Random Digit Dialing (RDD) techniques on both landlines and cell phones [26].

#### 2.1.2. Survey Design

States and territories utilize a probability sample of households with phones in their state, and all participating areas met this criterion for 2016 and 2018 [15]. Data are weighted to make the sample more representative of the population from which the data were collected [15]. The design weight considers the probability of selection and adjusts for non-response bias and non-coverage errors [15].

The annual questionnaire is currently utilized in all fifty states, the District of Columbia, and three U.S. territories and has advanced from a landline-only survey to include cellular phones [26]. These changes required BRFSS to change their statistical weighting methods in 2011 from “post-stratification” to an advanced weighting method called iterative proportional fitting or “raking” [27]. Advantages of these weighting methods means that BRFSS should better represent minority populations and varied income and education levels, reducing the likelihood of selection bias while increasing representation [27]. This change also means that the comparability of pre-2010 BRFSS datasets to post-2010 will change [27].

#### 2.1.3. Questionnaire

The BRFSS questionnaire consists of core questions (fixed and rotating), optional modules, and state-added questions. The fixed questions are asked yearly, and rotating core questions are asked every other year. Women’s health questions, which include cancer screening questions, are asked in even-numbered years [26].

#### 2.1.4. Inclusion Criteria

This study used BRFSS data for Arizona and New Mexico for 2016 and 2018, with 16,976 and 14,802 participants completing the survey interviews, respectively, for a total of 31,778 participants combined. The datasets were retrieved from the BRFSS website. BRFSS 2020 data were not included in this analysis due to the COVID-19 pandemic and its impact on access to healthcare and routine preventative screening, like mammograms, during the shelter-in-place ordinances and the overfilling of hospitals due to the infection.

**Figure 1:**
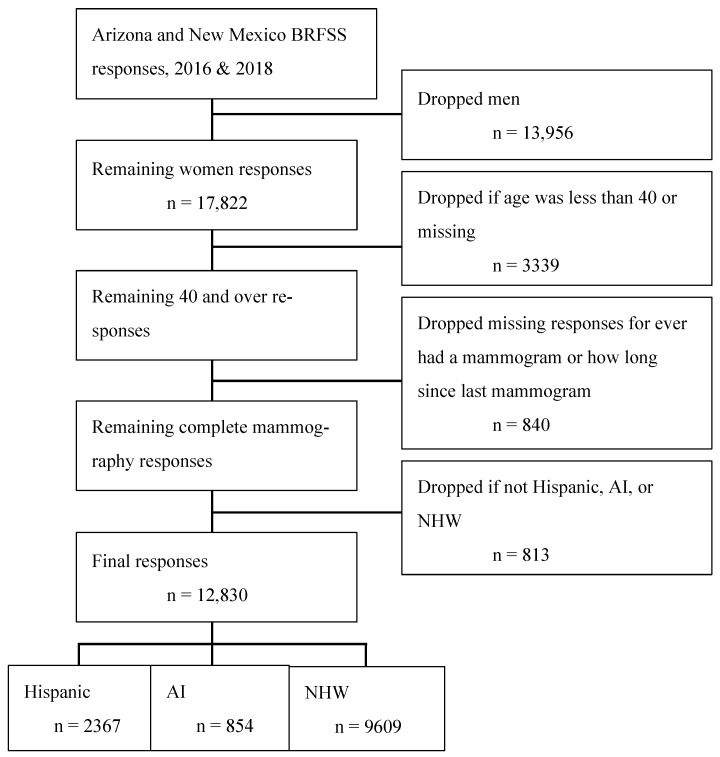
This figure shows the flow chart for all BRFSS participants in the two states for the two years and the impact of the exclusion criteria.

Inclusion criteria were women aged 40 or older who were Arizona or New Mexico residents. Exclusion criteria were eligible women with missing responses to the questions ‘Ever had a mammogram’. Also, women who did not identify as NHW, Hispanic, or AI were excluded. See Figure 1 below.

### 2.2. Measures

#### 2.2.1. Outcome or Measures of Breast Cancer Screening

The primary outcomes were: (1) ever having had a mammogram, (2) time since last mammogram (mutually exclusive responses). The responses for ever having had a mammogram were dichotomous (Yes/No). Missing and refused responses were excluded. Variables for mammography compliance recommendations were calculated utilizing the primary outcome responses. Age was categorized based on the breast cancer screening guidelines described in Table 1. Analyses were also conducted for women in the 40–49 age category, although the USPSTF guidelines currently consider screening before age 50 as an individual choice.

#### 2.2.2. Other Variables

Variables associated with health utilization and sociodemographic characteristics were selected as potential explanatory variables or confounders. These variables were selected based on prior knowledge or because they related to the Healthy People 2020 categories for social determinants of health [28]. Also, these variables had to be present in both Arizona and New Mexico BRFSS datasets. Final variables included: general health status, healthcare coverage, having a personal doctor, cost as an issue for not visiting a healthcare provider within the past 12 months, visiting a healthcare provider within the past 12 months, employment, education, age, education level, annual household income.

#### 2.2.3. Statistical Methods

Weighted frequencies and 95% confidence intervals (CI) were calculated for USPSTF mammography compliance by race/ethnicity group and age group. All analyses utilized the SVY command to account for the BRFFS complex survey design for each year and state. The _LLCPWT data weight was used for both Arizona and New Mexico for the 2016 and 2018 datasets. Logistic regression was used to calculate odds ratios (ORs) and 95% CI for USPSTF mammography compliance as the measure of effect. Adjusted OR (AOR) values were calculated with the following covariates: general health, healthcare coverage, personal doctor, cost issue for not visiting a healthcare provider within the past 12 months, visit to a healthcare provider within the past 12 months, employment, education, age, education level, and annual household income. Covariates were also checked for collinearity.

A sensitivity analysis that analyzed the adjusted models by state was conducted separately for Arizona and New Mexico to assess the consistency of the results by state. Descriptive statistics and mammography history tables by state are included in Supplementary Materials Table S1. STATA 17 was used for all statistical analyses.

## 3. Results

### 3.1. Characteristics of Participants

Overall, 12,830 women 40 years of age and older responded to the BRFSS in Arizona and New Mexico in 2016 and 2018 who met the inclusion criteria. Table 2 displays the sociodemographic characteristics of the women. NHW women comprised the largest percentage of the study population (74.9%), with 18.4% Hispanic women and 6.7% AI women. The population distribution by age was similar between race and ethnic categories. NHW had the highest percentage of college or technical school graduates (30.1%) compared to Hispanic and AI (~13% each). Employment for a business or self-employed was highest amongst AI (44.6%). BRFSS designated five income categories, and the distribution differed by racial and ethnic groups. Fewer Hispanic and AI women were in the $50,000 or more category (24.3% and 17.8%, respectively) than NHW women (51.7%). A higher percentage of Hispanics and AI reported not going to a doctor in the past 12 months due to cost (20.6% and 16.2%, respectively) compared to NHW (9.1%). About 75% of participant responses were from Arizona.

### 3.2. Breast Cancer Screening History

NHW women reported the highest frequency for ever having a mammogram (93.5%) when compared to Hispanic (85.7%) and AI (83.6%) women. Table 3 shows the time since last mammogram by the race/ethnic groups. Of those women who reported ever having had a mammogram, the percentages of women who reported mammography in the past year were similar for NHW and Hispanic (~54%) and slightly lower for AI (52.6%). Mammography in the past two years, but not in the past year, was highest amongst Hispanic women (23.3%), followed by AI (22.2%) and NHW (18.9%). A higher percentage of NHW women reported that their last mammogram was greater than 5 years ago (11.9%), compared to 7.2% of Hispanic women and 8.6% of AI women.

Table 3 also shows mammography guideline compliance based on the recommendations of three organizations. For the age group 40–49, NHW women had the highest percentage (57.2%) reporting a mammogram within the past two years compared to Hispanic and AI (54.5% and 45.3%, respectively). For women in the 50–74 age grouping, the frequency of reporting a mammogram within the past two years, and therefore compliant with USPSTF guidelines, was 75.1% for Hispanic women, 73.9% for NHW women, and 71.0% for AI women. The percentage of women who reported mammography compliance for the ACS guidelines was highest amongst NHW women (63.0%), followed by Hispanic (58.8%) and AI (53.8%). Since the recommendations for the ACS change by age, the age categories and time frames were reported separately to better understand compliance. Compliance for ACS was lower in the 40–54 category than the 55–80+ category across all racial and ethnic groups. Following ACS recommendations, Hispanic women in the 55–80+ age category surpassed NHW women for mammography in the past two years. As for ACR compliance, NHW women aged 40 and over had the highest percentage of reporting mammography within the past year (50.6%) compared to Hispanic (46.9%) and AI (43.9%) women. ACR compliance was the lowest (49.4%) when compared to both USPSTF (74.1%) and ACS (61.5%). An analysis by state for Arizona and New Mexico is included in Supplementary Materials Table S2. Furthermore, percentages of women 50–74 years who are USPSTF compliant by sociodemographic/health utilization variables are included in Supplementary Materials Table S3.

### 3.3. Factors Associated USPSTF Compliance-Adjusted Models

Table 4 shows the unadjusted and adjusted associations for women 50–74 years of age by USPSTF compliance (reporting a mammogram within the past two years) and various characteristics and behaviors. AI and NHW women who were screening-compliant had greater odds of reporting good to excellent health than non-compliant women, although the odds ratios were statistically significant only for NHW women (see Table 4). Compliance was significantly associated with having current medical insurance for all included racial and ethnic groups, but strikingly higher for AI women with an AOR = 9.6 (CI = 3.7–25.0). Mammography compliance also was positively associated with having a personal doctor for Hispanic and NHW women (AOR 1.6 CI = 0.8–3.2 and AOR = 2.8 CI = 2.0–3.9, respectively), but not for AI women (AOR = 0.6 CI = 0.3–1.4).

Furthermore, mammography compliance was strongly associated with reporting having seen a doctor in the past 12 months for all race/ethnic groups, with the effect strongest for Hispanic women (AOR = 4.2 CI = 2.4–7.2). Cost being an issue for not seeing a doctor in the past 12 months and current employment or retired status were modestly associated with mammography compliance for Hispanic and NHW women, but no association was observed for AI women. Mammography-compliant Hispanic and NHW women were 1.5 and 1.2 times as likely to report being employed (not statistically significant), while current employment status was not a factor for AI women.

Adjusted odds ratios for compliance for women were higher for those who were retired than non-compliant women. For instance, Hispanic women who reported being retired were two times as likely to be mammography-compliant (AOR = 2.0, CI = 1.1, 3.6). There was no clear trend for compliance by age category, although, Hispanic and AI women aged 55 through 69 had higher odds of having had a mammogram in the past two years.

AI women who graduated at least high school had greater odds of reporting a mammogram within the past two years, with no association observed for Hispanic and NHW women. AI and NHW women who reported having an annual household income of $50,000 or more had greater odds of mammography in the past two years (AOR = 1.8 CI = 0.8–4.0 and AOR = 1.4 CI = 1.1–1.8, respectively).

### 3.4. Sensitivity Analyses

A sensitivity analysis was done to identify whether similar effects were seen between the two states. Table 5 shows the adjusted associations between women 50–74 years of age reporting a mammogram within the past two years by various characteristics and behaviors for Arizona and New Mexico, respectively. There were few differences between the associations observed for the pooled data and the separate states for reporting good to excellent health, having current medical insurance, having a personal doctor, education, or employment.

However, for AI women, differences in Arizona and New Mexico were seen for having visited a doctor in the past 12 months (AOR = 1.1 and 6.7, respectively) and retirement status (OR = 0.9 and 1.8, respectively). Furthermore, the AOR was 30.4 for Arizona AI women for the association with current medical insurance compared to AOR = 3.6 for New Mexico AI women and AOR = 9.6 for the pooled data. These odds ratios indicate potential effect modification, although the confidence intervals overlap. This finding may suggest the need to understand whether these differences may be due to policy issues or how the questions were asked in each state. There was state variation by race/ethnicity for the association between mammography compliance and having visited a doctor in the past 12 months. The associations were strong for all groups in New Mexico, but the association for AI women in Arizona was slight (AOR = 1.1) compared to AI women in New Mexico (AOR = 6.7). The width of confidence intervals suggest that these differences may be due to sample size issues.

## 4. Discussion

This study utilized BRFSS data from 2016 and 2018 for Arizona and New Mexico to identify factors associated with women getting timely mammography screening. NHW women had the highest percentage of reporting they ever had a mammogram, followed by Hispanic and AI women, respectively. However, Hispanic women had the highest proportion who adhered to the USPSTF within the 50–74 age group. These findings support research published from Benavidez in 2021 that reported Hispanic women in the U.S. had higher breast cancer screening rates than NHW [28].

Overall, compliance with the USPSTF recommendations was highest compared to the ACS and ACR. This finding makes sense because the USPSTF two-year timeframe is more forgiving than the stringent 1-year recommendations of the other two organizations.

The adjusted odds ratios suggest that current medical insurance was positively associated with USPSTF compliance for Hispanic, AI, and NHW women aged 50–74. These findings support conclusions described by various publications that all found that women who did not have current medical insurance were less likely to have had a mammogram within the past two years for all women and specifically for AI and Hispanic women [18,21,22,28]. According to the U.S. Department of Health and Human Services (HHS) Office of Minority Health (OMH), 18.7% and 14.9% of Hispanic and AI/AN did not have healthcare coverage in 2019, respectively, as compared to NHW at 6.3% [29,30]. Hispanics had the highest uninsured rate amongst all U.S. racial and ethnic groups; of the Hispanic subgroups, 20.3% of Mexicans did not have coverage [29]. This disparity was reflected in the number of women the NBCCEDP served in Arizona and New Mexico between 2016 and 2021. Of the women supported by the Arizona and New Mexico NBCCEDP, 80.2% and 82.3% were Hispanic in the Arizona and New Mexico programs, and 0.6% were AI in Arizona, compared to 9.7% in New Mexico [31]. Possible reasons behind this difference among the AI population by state could be because there is a higher number of AI women in New Mexico and, of those in New Mexico, more quality for NBCCEDP services. Another might be greater accessibility to the NBCCEDP locations in New Mexico than for Arizona AI women.

Although we could not differentiate between the type of care those individuals with current medical insurance received, a previous study found that AI who reported using IHS fared better than AI individuals who do not have any coverage [32]. Nonetheless, AI still experienced gaps in care when compared to insured NHW [32]. These results highlight the continued barriers that access to care can have on mammography compliance. The sensitivity analysis showed some state variability for the association between having visited a doctor in the past 12 months and retirement for AI women (Arizona AOR = 1.1 vs. New Mexico AOR = 6.7 and Arizona AOR = 0.9 and New Mexico AOR = 1.8, respectively). This difference in the AI population could point to Arizona having more barriers to having current medical insurance and visiting a doctor in the past 12 months compared to New Mexico.

Having a personal doctor or healthcare provider also increased the odds of mammography compliance for Hispanic (AOR = 1.6) and NHW women (AOR = 2.8), which was consistent for both states. Building trust with a healthcare provider takes time, and more respect and trust between a provider and patient can positively impact the patient’s receptiveness to their provider’s suggestions [33]. We found that over 36% of New Mexico AI women received healthcare from IHS. However, this question was not asked on the Arizona BRFSS questionnaires. IHS has a high turnover rate for doctors and healthcare providers, so it is unlikely that an AI patient would have a consistent doctor over a long period. Additionally, findings from the Adakai et al. study report that as the AI/AN population has become younger and more racially diverse, larger numbers of AI/AN are residing in cities, limiting continuity of care through IHS and, possibly, the ability of AI/AN to obtain and retain a personal doctor or healthcare provider [34]. Therefore, the findings of AI women without a personal doctor being associated with lower mammography make sense. A previous analysis using 2017 BRFSS data also revealed that the prevalence of AI having a personal doctor was lower than that of NHW [34].

A visit to a healthcare provider in the past 12 months is a preventative healthcare service that can enhance health promotion. Providers can use this opportunity to promote cancer screening to their patients based on their risk level, age, and family history [10]. Although Orji et al. cited literature that supported that routine checkups were associated with breast cancer screening for White, Black, and Hispanic women in the U.S., our findings support that having visited a doctor in the past year was strongly and positively associated with having had a mammogram within the past two years for all included racial and ethnic groups, with AI women having the highest odds ratio. Interestingly, the Orji et al. study, which included data from 2017 through 2019, found that AI women received no benefit [10]. However, this study did not look at geographic variation.

Unsurprisingly, the cost of seeing a doctor was a barrier for Hispanic and NHW women in obtaining mammograms. However, the sensitivity analysis showed that cost impacted Hispanic women living in Arizona versus New Mexico differently (AOR = 1.3 and =0.7, respectively). More Hispanic women in New Mexico responded “yes” to having health insurance, yet the cost of healthcare seemed to be more of an issue in New Mexico. Possible reasons for this could be higher premium or co-pay costs, although this was not assessed in this study. However, it was interesting that cost was not an issue for AI. This finding could be because IHS enrollment reduced the disparity by increasing healthcare access through primary care and screening services. This potential explanation was described by Burnett-Hartman et al. when they noted that IHS enrollment decreased the gap in the proportion of patients with late-stage cancer diagnoses between AI/ANs and NHWs [23]. Hispanic and NHW women reporting current employment had greater odds of having had a mammogram in the past two years, but this variable did not impact AI as greatly. Again, AI enrollment in IHS could explain why employment status would have less or little bearing on access to care. Mammography compliance was associated with retirement status for all included racial and ethnic groups, with the highest odds ratio seen for Hispanic women (OR = 1.9). This finding could be due to these women being more likely to be eligible for Medicare, which increased access to preventative services that individuals may not have had access to previously.

Although it was anticipated that there would be stronger positive associations between socioeconomic factors like current employment, college/technical school graduation, and annual income over $50,000 across all included racial and ethnic groups, there did not appear to be for these populations. Employment impacted Hispanic and NHW, but not AI women. College or technical school graduation impacted AI, but not Hispanic and NHW women. Annual household income of $50,000 or more impacted AI and NHW, but not Hispanic women. These findings differed from those reported by Benavidez et al., who found that current employment, income over $50,000, and increased age positively impacted USPSTF compliance [28]. In the Benavidez study, there was no difference in meeting USPSTF guidelines between those with a college or technical school education and those without one [28]. However, Benavidez did not stratify by racial and ethnic groups [28]. When AI was included, the findings grouped AI in the “Other” group with Asian, Hawaiian, and Pacific Islander women, similar to the work of Orji [28]. These findings support the need to look at AI separately in studies to better understand associations that impact this underrepresented racial group.

Potential limitations are those inherent within these self-reported BRFSS data, including recall bias and the variable sample sizes for the different racial and ethnic groups. Although the proportions could be seen as representative of the state’s populations, the smaller sample size for the AI group meant lower power to identify statistically significant effects. In 2017, the U.S. Department of Health and Human Services (HHS) Office of Minority Health (OMH) partnered with the CDC to conduct additional BRFSS interviews in 11 states, including Arizona, to improve the understanding of the health status of AI and Alaska Native (AN) communities. However, this increased sampling was only for the 2017 interview periods, and the cancer screening questions were not included in this larger study sample [35]. This initiative did, however, recognize that sampling is an issue and that increased sampling is likely needed to improve the health data for Native communities. Another problem for this analysis is that the BRFSS does not confirm whether an individual is an enrolled member of a tribal nation when interviewing participants who respond that they are AI/AN. Therefore, since BRFSS does not ask about tribal enrollment, it may not be representative of the tribes in each state.

## 5. Conclusions

During 2016 to 2018, 85.7% (CI 82.4–88.5) of Hispanic and 83.6% (CI 79.5–87.0) of AI women reported ever receiving a mammogram, compared to 93.5% (CI 92.5–94.3) of NHW women. Overall, 75.1% of Hispanic women 50–74 years of age reported a mammogram in the past two years (USPSTF compliant) compared to 73.9% of NHW and 71.0% of AI women. Hispanic women had the highest USPSTF compliance compared to AI and NHW, consistent with other literature [28]. Screening compliance of Southwestern Hispanic and AI women is influenced by age, and women aged 50–74 are more likely to comply with the two-year mammography USPSTF criteria than the age groups 40–54 and 75+. As age increased by 5-year increments in the adjusted model, so did the odds of mammography compliance for Hispanic and AI women up to age 69. Factors positively associated with USPSTF breast cancer screening compliance for Hispanic and AI women were current medical insurance, visiting a healthcare provider in the past 12 months, and retirement. Future studies should include comparisons between rural- and urban-residing individuals as this might help explain some differences between the states and race/ethnic groups. Because of the small sample size of AI in both states’ datasets, other research recruitment strategies may be needed to have more accurate results. For instance, the 2017 enhanced sample size used for AI would be appropriate for additional years to explore cancer-related questions further. Additionally, the COVID-19 pandemic created barriers to women’s health screening as elective and non-life-threatening treatments were paused because of the overwhelmed healthcare system. This period should be investigated to determine how it impacted screening and if it continues to impact screening for women of this demographic. Planned future analysis will explore current BRFSS data and include all racial and ethnic groups.

## Figures and Tables

**Table 1:** Current breast cancer screening guidelines for average-risk women by age groups from three different organizations.

Age Groupings	United States Prevention Services Task Force (USPSTF) [13]	American Cancer Society (ASC) [12]	American College of Radiology (ACR) [16]
40 to 49 years with average risk	Individual choice	40–44, individual choice	Once a year
		45–54, once a year	
50 to 74 years with average risk	Once every two years		Once a year
		55–74, once every two years	
75 years or older with average risk	Current evidence is insufficient	Continue screening if in good health and have a life expectancy of 10+ years	Decision should be based on health status rather than age

**Table 2:** Demographic characteristics ^a^ of women 40 years of age and older for selected racial/ethnic groups, Arizona and New Mexico, BRFSS, 2016 and 2018.

	All	Hispanic	AI	NHW
	*n* = 12,830	*n* = 2367	*n* = 854	*n* = 9609
	% (95% CI)	% (95% CI)	% (95% CI)	% (95% CI)
Age, years				
40–49	22.9 (21.5, 24.4)	35.0 (31.4, 38.8)	28.3 (24.0, 33.1)	18.1 (16.7, 19.7)
50–74	61.0 (59.4, 62.5)	57.0 (53.2, 60.7)	63.9 (58.9, 68.5)	62.3 (60.4, 64.0)
75–80+	16.1 (15.2, 17.1)	8.0 (6.6, 9.6)	7.8 (5.5, 11.0)	19.7 (18.4, 20.8)
Marital status				
Married	55.4 (53.9, 57.0)	56.0 (52.3, 59.6)	36.8 (32.3, 41.6)	56.2 (54.6, 57.9)
Veteran				
Yes	2.2 (1.8, 2.6)	1.8 (1.1, 3.1)	1.5 (0.9, 2.6)	2.4 (1.9, 2.9)
Level of education completed				
Did not graduate high school	15.5 (14.1, 17.0)	40.9 (37.0, 44.9)	22.3 (18.2, 27.0)	5.8 (4.9, 6.7)
Graduated high school	23.0 (21.8, 24.2)	23.6 (21.0, 26.5)	30.5 (26.3, 35.2)	22.4 (21.0, 23.8)
Attended college/technical school	36.7 (35.2, 38.2)	23.4 (20.6, 26.4)	33.7 (29.3, 38.4)	41.8 (40.1, 43.5)
Graduated college/technical school	24.8 (23.7, 25.9)	12.1 (10.3, 14.1)	13.5 (10.6, 17.0)	30.1 (28.8, 31.5)
Employment status				
Currently not working	28.6 (27.1, 30.1)	41.7 (38.0, 46.6)	35.1 (30.1, 39.9)	23.4 (21.9, 24.9)
Employed for wages or self employed	37.3 (35.8, 38.8)	39.8 (36.3, 43.5)	44.6 (39.7, 49.5)	36.0 (34.3, 37.7)
Retired	34.1 (32.8, 35.5)	18.5 (16.2, 20.9)	20.4 (16.8, 24.6)	40.6 (39.1, 42.2)
Income				
Less than $15,000	13.1 (11.9, 14.4)	24.8 (21.1, 28.8)	33.3 (28.5, 38.4)	7.7 (6.9, 8.6)
$15,000 to >$25,000	19.9 (18.5, 21.3)	29.4 (25.8, 33.2)	27.9 (23.6, 32.6)	15.9 (14.6, 17.3)
$25,000 to >$35,000	10.9 (9.9, 11.9)	11.3 (9.2, 13.8)	10.3 (7.6, 14.8)	10.7 (9.7, 11.8)
$35,000 to >$50,000	12.9 (11.9, 14.0)	10.3 (8.2, 12.9)	10.8 (7.7, 14.8)	14.0 (12.8, 15.2)
$50,000 or more	43.3 (41.6, 44.9)	24.3 (21.2, 27.6)	17.8 (14.1, 22.1)	51.7 (49.9, 53.5)
Last visited a doctor for a routine checkup				
Within past year	79.0 (77.8, 80.2)	75.7 (72.4, 78.8)	75.4 (70.1, 79.7)	80.4 (79.1, 81.7)
5 or more years ago	4.0 (3.4, 4.6)	3.5 (2.3, 5.3)	4.5 (2.7, 7.4)	4.2 (3.6, 4.8)
Cost an issue for not visiting doctor in past 12 months				
Yes	12.4 (11.3, 13.5)	20.6 (17.6, 23.9)	16.2 (13.0, 20.0)	9.1 (8.2, 10.1)
State				
Arizona	75.8 (75.2, 76.4)	62.3 (59.3, 65.1)	53.9 (49.3, 58.4)	81.9 (81.3, 82.6)
New Mexico	24.4 (23.6, 24.9)	37.8 (34.9, 40.7)	46.1 (41.6, 50.7)	18.1 (17.4, 18.8)

^a^ Weighted percentages using CDC final weights.

**Table 3:** Percentage ^a^ of women 40 years of age and older by racial/ethnic group by their mammography history: Arizona and New Mexico, BRFSS, 2016 and 2018.

	All	Hispanic	AI	NHW
	*n* = 12,830	*n* = 2367	*n* = 854	*n* = 9609
	% (95% CI)	% (95% CI)	% (95% CI)	% (95% CI)
Ever had a mammogram				
Yes	91.1 (90.0, 92.1)	85.7 (82.4, 88.5)	83.6 (79.5, 87.0)	93.5 (92.5, 94.3)
How long since last mammogram (mutually exclusive)				
Within the past year	54.2 (52.6, 55.8)	54.7 (50.8, 58.6)	52.6 (47.3, 57.8)	54.1 (52.4, 55.8)
Within the past 2 years	20.1 (18.8, 21.5)	23.3 (19.9, 27.2)	22.2 (18.2, 26.8)	18.9 (17.5, 20.4)
Within the past 3 years	8.7 (7.9, 9.6)	7.8 (6.1, 9.9)	11.2 (8.2, 15.0)	8.9 (8.0, 10.0)
Within the past 5 years	6.3 (5.6, 7.1)	7.0 (5.3, 9.1)	5.5 (3.4, 8.6)	6.1 (5.4, 6.9)
Five or more years	10.7 (9.8, 11.6)	7.2 (5.6, 9.4)	8.6 (5.8, 12.4)	11.9 (10.9, 13.1)
U.S. Preventative Services Task Force (USPSTF) Compliant				
40–49, mammogram in past 2 years	55.6 (51.7, 59.4)	54.5 (47.4, 61.3)	45.3 (36.1, 54.9)	57.2 (52.5, 61.9)
50–74, mammogram in past 2 years	74.1 (72.4, 75.7)	75.1 (70.7, 79.0)	71.0 (65.2, 76.2)	73.9 (72.1, 75.7)
75–80+, mammogram in past 2 years	60.6 (57.6, 63.6)	63.0 (54.6, 70.7)	55.5 (37.6, 72.1)	60.4 (57.1, 63.6)
American Cancer Society (ACS) Compliant				
Overall ACS compliance for 40+	61.5 (60.0, 63.0)	58.8 (55.1, 62.5)	53.8 (48.8, 58.6)	63.0 (61.3, 64.6)
40–54, mammogram within past year	43.6 (40.7, 46.6)	41.1 (35.8, 46.6)	31.5 (25.3, 38.4)	46.2 (42.5, 49.9)
55–80+, mammogram in past 2 years	71.7 (70.2, 73.1)	77.2 (73.5, 80.6)	71.9 (66.1, 77.1)	70.3 (68.6, 71.9)
American College of Radiology (ACR) Compliant				
40–80+, mammogram within past year	49.4 (47.8, 50.9)	46.9 (43.2, 50.6)	43.9 (39.2, 48.8)	50.6 (48.9, 52.2)

^a^ Weighted percentages using CDC final weights.

**Table 4:** Adjusted odd ratios for women aged 50–74 for associations self-reported mammography in the past two years and sociodemographic/health utilization variables by racial/ethnic group, 2016 and 2018.

	Hispanic	AI	NHW
	*n* = 1484	*n* = 600	*n* = 6161
	AOR (95% CI)	AOR (95% CI)	AOR (95% CI)
General health			
Poor to Fair	1	1	1
Good to Excellent	0.9 (0.5, 1.6)	1.2 (0.6, 2.3)	1.3 * (1.0, 1.7)
Current medical insurance			
No	1	1	1
Yes	2.0 (0.9, 4.4)	9.6 * (3.7, 25.0)	1.9 * (1.1, 3.2)
Personal doctor or health care provider			
No	1	1	1
Yes	1.6 (0.8, 3.2)	0.6 (0.3, 1.4)	2.8 * (2.0, 3.9)
Visited health care provider in past 12 months			
No	1	1	1
Yes	4.2 * (2.4, 7.2)	2.9 * (1.4, 5.6)	3.2 * (2.5, 4.1)
Cost an issue for not visiting doctor in past 12 months			
No	1	1	1
Yes	0.9 (0.5, 1.7)	1.1 (0.5, 2.5)	0.8 (0.5, 1.1)
Employment status			
Not working	1	1	1
Employed	1.5 (0.8, 3.0)	0.9 (0.4, 1.8)	1.2 (0.9, 1.6)
Retired	2.0 (1.1, 3.6)	1.2 (0.5, 2.7)	1.3 (1.0, 1.8)
Age			
50–54	1	1	1
55–59	2.6 (1.2, 5.4)	1.5 (0.6, 3.6)	0.7 (0.5, 1.1)
60–64	1.6 (0.7, 3.5)	2.2 (1.0, 4.9)	0.9 (0.7, 1.3)
65–69	1.6 (0.7, 3.5)	1.8 (0.7, 4.7)	0.9 (0.6, 1.4)
70-74	1.1 (0.4, 2.7)	0.6 (0.2, 1.6)	0.6 * (0.4, 0.9)
Graduated college/technical school			
No	1	1	1
Yes	1.0 (0.5, 2.0)	1.4 (0.7, 3.0)	0.9 (0.7, 1.2)
Annual household income $50,000 or more			
No	1	1	1
Yes	0.9 (0.4, 2.0)	1.8 (0.8, 4.0)	1.4 * (1.1, 1.8)

Reference group: women respondents aged 50–74 who have had a mammogram in the past two years. * Represents statistical significance *p* < 0.05. AOR models adjusted for all other variables in the table.

**Table 5:** Adjusted associations between self-reported mammography in the past two years for women aged 50–74 living in Arizona and New Mexico and sociodemographic/health utilization variables by racial/ethnic group, 2016 and 2018.

	Hispanic				AI		NHW		
	Combined	Arizona	New Mexico	Combined	Arizona	New Mexico	Combined	Arizona	New Mexico
	*n* = 1484	*n* = 545	*n* = 939	*n* = 600	*n* = 306	*n* = 294	*n* = 6161	*n* = 4090	*n* = 2071
	AOR (95% CI)	AOR (95% CI)	AOR (95% CI)	AOR (95% CI)	AOR (95% CI)	AOR (95% CI)	AOR (95% CI)	AOR (95% CI)	AOR (95% CI)
General Health									
Poor to Fair	1	1	1	1	1	1	1	1	1
Good to Excellent	0.9 (0.5, 1.6)	0.8 (0.3, 2.1)	1.0 (0.6, 1.7)	1.2 (0.6, 2.3)	1.3 (0.5, 3.5)	1.3 (0.6, 2.9)	1.3 * (1.0, 1.7)	1.3 (0.9, 1.7)	1.5 * (1.0, 2.3)
Current medical insurance									
No	1	1	1	1	1	1	1	1	1
Yes	2.0 (0.9, 4.4)	2.2 (0.6, 7.4)	2.0 (0.7, 5.4)	9.6 * (3.7, 25.0)	30.4 * (6.9, 134.1)	3.6 * (1.1, 12.0)	1.9 * (1.1, 3.2)	1.6 (0.9, 2.9)	5.8 * (1.9, 17.6)
Personal doctor or health care provider									
No	1	1	1	1	1	1	1	1	1
Yes	1.6 (0.8, 3.2)	2.1 (0.7, 6.1)	1.4 (0.7, 2.9)	0.6 (0.3, 1.4)	0.7 (0.2, 2.4)	1.0 (0.4, 2.6)	2.8 * (2.0, 3.9)	2.8 * (1.9, 4.2)	2.7 * (1.7, 4.2)
Visited health care provider in past 12 months									
No	1	1	1	1	1	1	1	1	1
Yes	4.2 * (2.4, 7.2)	5.2 * (2.0, 13.8)	3.9 * (2.4, 6.5)	2.9 * (1.4, 5.6)	1.1 (0.4, 3.5)	6.7 * (2.9, 15.4)	3.2 * (2.5, 4.1)	3.2 * (2.3, 4.3)	3.3 * (2.4, 4.6)
Cost an issue for not visiting doctor in past 12 months									
No	1	1	1	1	1	1	1	1	1
Yes	0.9 (0.5, 1.7)	1.3 (0.5, 3.5)	0.7 (0.4, 1.3)	1.1 (0.5, 2.5)	1.1 (0.3, 3.1)	1.2 (0.5, 2.8)	0.8 (0.5, 1.1)	0.7 (0.4, 1.1)	0.9 (0.6, 1.6)
Employment status									
Not working	1	1	1	1	1	1	1	1	1
Employed	1.5 (0.8, 3.0)	1.4 (0.5, 3.9)	1.6 (0.9, 3.0)	0.9 (0.4, 1.8)	0.8 (0.2, 2.9)	0.8 (0.3, 1.9)	1.2 (0.9, 1.6)	1.2 (0.8, 1.7)	1.0 (0.7, 1.6)
Retired	2.0 (1.1, 3.6)	1.9 (0.7, 4.9)	2.2 * (1.1, 4.3)	1.2 (0.5, 2.7)	0.9 (0.3, 3.1)	1.8 (0.7, 5.2)	1.3 (1.0, 1.8)	1.3 (0.9, 1.9)	1.3 (0.8, 2.0)
Age									
50–54	1	1	1	1	1	1	1	1	1
55–59	2.6 (1.2, 5.4)	5.0 * (1.5, 16.3)	1.0 (0.5, 1.9)	1.5 (0.6, 3.6)	1.7 (0.5, 6.4)	1.1 (0.4, 3.2)	0.7 (0.5, 1.1)	0.8 (0.5, 1.2)	0.6 (0.4, 1.0)
60–64	1.6 (0.7, 3.5)	1.8 (0.5, 5.9)	1.3 (0.6, 2.7)	2.2 (1.0, 4.9)	2.3 (0.6, 7.8)	3.4 (0.9, 12.3)	0.9 (0.7, 1.3)	1.0 (0.6, 1.5)	0.8 (0.5, 1.3)
65–69	1.6 (0.7, 3.5)	1.8 (0.6, 5.9)	1.4 (0.6, 3.0)	1.8 (0.7, 4.7)	1.6 (0.4, 6.2)	1.5 (0.5, 4.0)	0.9 (0.6, 1.4)	1.1 (0.7,1.7)	0.5 * (0.3, 0.9)
70–74	1.1 (0.4, 2.7)	1.7 (0.4, 7.4)	0.6 (0.2, 1.4)	0.6 (0.2, 1.6)	0.9 (0.1, 5.0)	0.3 (0.1, 1.1)	0.6 * (0.4, 0.9)	0.6 (0.4, 1.0)	0.5 * (0.3, 0.9)
Graduated college/technical school									
No	1	1	1	1	1	1	1	1	1
Yes	1.0 (0.5, 2.0)	1.6 (0.4, 6.0)	0.6 (0.3, 1.0)	1.4 (0.7, 3.0)	1.0 (0.4, 2.9)	2.1 (0.8, 5.2)	0.9 (0.7, 1.2)	0.9 (0.7, 1.3)	1.0 (0.7, 1.3)
Annual household income $50,000 or more									
No	1	1	1	1	1	1	1	1	1
Yes	0.9 (0.4, 2.0)	0.7 (0.2, 2.8)	1.4 (0.7, 2.5)	1.8 (0.8, 4.0)	3.0 (0.7, 12.6)	1.2 (0.4, 3.4)	1.4 * (1.1, 1.8)	1.3 (1.0, 1.8)	1.7 * (1.2, 2.3)

Reference group: women respondents aged 50–74 who have had a mammogram in the past two years. * Represents statistical significance *p* < 0.05. AOR models adjusted for all other variables in the table.

## Data Availability

These data were downloaded from public information and can be accessed from the CDC website at https://www.cdc.gov/brfss/index.html accessed on 17 February 2022. Recoding schemes are available upon request.

## Supplementary Materials

The following supporting information can be downloaded at: https://www.mdpi.com/article/10.3390/ijerph21010019/s1, Table S1. Demographic characteristics ^a^ of women 40 years of age and older for selected racial/ethnic groups, by state for Arizona and New Mexico BRFSS, 2016 and 2018; Table S2. Percentage ^a^ of women 40 years of age and older by racial/ethnic group by their mammography history, by state for Arizona and New Mexico, BRFSS, 2016 and 2018; Table S3. Percentage ^a^ of women 50–74 years who are USPSTF compliant by sociodemographic/health utilization variables, Arizona and New Mexico, BRFSS, 2016 and 2018.
