# Supplementary material for: Mammography Compliance for Arizona and New Mexico Hispanic and American Indian Women 2016–2018
Source: Int J Environ Res Public Health. 2023 Dec 22;21(1):19. doi: 10.3390/ijerph21010019 (PMC10815889; doi:10.3390/ijerph21010019)
Supplement: Supplementary file 1 [file ijerph-21-00019-s001.zip › ijerph-2778668-supplementary.pdf]

Table S1. Demographic characteristics[a] of women 40 years of age and older for selected racial/ethnic groups, by state for Arizona and New Mexico BRFSS, 2016 and 2018.

	All		Hispanic		AI		NHW	
	Arizona<br>n= 7,806<br>% (95% CI)	New Mexico<br>n= 5,024<br>% (95% CI)	Arizona<br>n= 868<br>% (95% CI)	New Mexico<br>n= 1,499<br>% (95% CI)	Arizona<br>n= 422<br>% (95% CI)	New Mexico<br>n= 432<br>% (95% CI)	Arizona<br>n= 6,516<br>% (95% CI)	New Mexico<br>n= 3,093<br>% (95% CI)
Age, years								
40–49	22.8 (21.0, 24.7)	23.3 (21.6, 25.1)	37.6 (32.2, 43.4)	30.8 (27.6, 34.1)	26.9 (21.0, 33.9)	29.9 (24.1, 36.5)	18.5 (16.8, 20.3)	16.6 (14.6, 18.7)
50–74	60.9 (58.9, 62.8)	61.4 (59.4, 63.3)	56.1 (50.5, 61.7)	44.0 (40.6, 47.4)	65.9 (58.7, 72.5)	61.5 (54.8, 67.7)	62.0 (60.1, 64.0)	63.6 (61.1, 66.1)
75–80+	16.4 (15.2, 17.6)	15.4 (14.1, 16.8)	6.3 (4.6, 8.5)	10.8 (9.0, 12.9)	7.2 (4.1, 12.4)	8.6 (5.6, 13.0)	19.5 (18.1, 21.0)	19.8 (17.9, 21.9)
Marital status								
Married	55.9 (54.0, 57.8)	54.1 (52.1, 56.1)	57.7 (52.2, 63.2)	53.0 (49.6, 56.5)	36.0 (29.4, 43.1)	37.8 (32.0, 44.0)	56.0 (54.1, 58.0)	57.1 (54.6, 59.6)
Veteran								
Yes	2.3 (1.9, 2.9)	1.8 (1.4, 2.3)	2.1 (1.1, 4.2)	1.3 (0.7, 2.4)	2.2 (1.2, 4.2)	0.7 (0.3, 1.5)	2.4 (1.9, 3.0)	2.3 (1.7, 3.0)
Level of education completed								
Did not graduate high school	14.8 (13.0, 16.7)	17.7 (16.3, 19.5)	45.6 (39.9, 51.5)	33.0 (29.8, 36.4)	27.8 (18.4, 32.4)	19.4 (15.1, 24.6)	5.7 (4.7, 6.8)	5.9 (4.7, 7.4)
Graduated high school	23.0 (21.5, 24.5)	23.1 (21.5, 24.7)	22.1 (18.3, 26.5)	26.2 (23.4, 29.1)	31.6 (25.4, 38.5)	29.3 (23.8, 35.5)	22.9 (21.4, 24.6)	19.9 (18.0, 21.9)
Attended college/technical school	37.6 (35.8, 39.5)	33.8 (31.9, 35.7)	20.7 (16.9, 25.1)	27.9 (24.7, 31.3)	30.8 (24.8, 37.5)	37.1 (31.1, 43.6)	42.6 (40.7, 44.7)	37.8 (35.4, 40.4)
Graduated college/technical school	24.6 (23.3, 26.1)	25.3 (23.8, 26.9)	11.6 (9.0, 14.7)	12.9 (11.1, 15.0)	12.9 (9.1, 17.9)	14.2 (10.2, 19.4)	28.7 (27.2, 30.3)	36.4 (34.2, 38.7)
Employment status								
Currently not working	28.3 (26.5, 30.3)	29.4 (27.6, 31.2)	44.3 (38.6, 50.1)	37.5 (34.3, 40.8)	35.7 (29.2, 42.7)	34.3 (28.7, 40.4)	23.6 (21.9, 25.4)	22.5 (20.4, 24.7)
Employed for wages or self employed	36.6 (34.8, 38.5)	39.4 (37.5, 41.3)	39.4 (34.0, 44.9)	40.6 (37.3, 44.0)	41.9 (34.8, 49.2)	47.7 (41.3, 54.2)	35.7 (33.8, 37.6)	37.4 (35.0, 39.9)
Retired	35.0 (33.4, 36.7)	31.2 (29.5, 32.9)	16.3 (13.3, 20.0)	21.9 (19.4, 24.7)	22.4 (17.1, 28.9)	18.0 (13.6, 23.4)	40.7 (38.9, 42.6)	40.1 (37.8, 42.5)
Income								
Less than $15,000	11.9 (10.3, 13.6)	16.8 (15.3, 18.4)	26.2 (20.5, 32.7)	22.7 (19.8, 25.8)	39.6 (32.2, 47.6)	26.2 (21.2, 31.8)	7.0 (6.0, 8.0)	10.9 (9.3, 12.6)
$15,000 to >$25,000	18.7 (17.0, 20.6)	23.2 (21.4, 25.0)	28.4 (23.0, 34.6)	30.8 (27.4, 34.3)	24.0 (18.3, 30.7)	32.3 (26.2, 39.1)	15.9 (14.4, 17.6)	16.0 (14.2, 18.0)
$25,000 to >$35,000	10.6 (9.5, 11.9)	11.6 (10.3, 13.0)	10.9 (7.8, 15.0)	11.9 (9.8, 14.4)	9.0 (5.8, 13.6)	11.8 (7.7, 17.8)	10.6 (9.5, 11.9)	11.2 (9.5, 13.2)
$35,000 to >$50,000	13.2 (11.9, 14.5)	12.2 (10.9, 13.6)	11.0 (7.9, 15.1)	9.4 (7.5, 11.8)	7.2 (3.9, 12.9)	14.8 (10.0, 21.1)	14.0 (12.6, 15.4)	13.9 (12.2, 15.8)
$50,000 or more	45.6 (43.5, 47.7)	36.4 (34.4, 38.4)	23.6 (19.1, 28.8)	25.3 (22.3, 28.5)	20.2 (14.7, 27.2)	15.0 (10.8, 20.3)	52.5 (50.4, 54.7)	48.0 (45.4, 50.6)
Current medical insurance								
Yes	92.3 (90.9, 93.5)	93.5 (92.4, 94.4)	76.9 (71.6, 81.5)	88.5 (86.1, 90.5)	89.4 (82.9, 93.6)	94.2 (90.8, 96.4)	96.7 (95.9, 97.4)	97.2 (96.2, 97.9)
Last visited a doctor for a routine checkup								
Within past year	80.4 (78.8, 81.9)	74.7 (72.9, 76.3)	76.5 (71.4, 81.0)	74.4 (71.3, 77.3)	74.3 (66.7, 80.7)	76.7 (70.7, 81.8)	81.7 (80.2, 83.1)	74.6 (72.3, 76.7)
5 or more years ago	3.9 (3.2, 4.7)	4.3 (3.7, 5.1)	3.6 (1.9, 6.7)	3.3 (2.4, 4.5)	4.8 (2.2, 10.2)	4.3 (2.5, 7.2)	3.9 (3.3, 4.7)	5.1 (4.2, 6.3)
Cost an issue for not visiting doctor in past 12 months								
Yes	12.3 (11.0, 13.8)	12.5 (11.3, 13.5)	22.9 (18.5, 28.1)	16.8 (14.4, 19.5)	13.3 (9.4, 18.5)	19.5 (14.7, 25.3)	9.3 (8.2, 10.5)	8.3 (7.0, 9.8)
Primary source of health care coverage								
Employer		35.2 (33.2, 37.2)		35.9 (32.4, 39.5)		16.9 (12.4, 22.5)		37.1 (34.6, 39.7)
Medicare		31.8, 30.0, 33.6)		28.3 (25.3, 31.6)		20.1 (15.1, 26.2)		35.8 (33.4, 38.2)
Medicaid or state program		15.6 (14.1, 17.2)		23.4 (20.4, 26.6)		21.6 (16.2, 28.3)		9.4 (7.9, 11.2)
IHS or Tribal Health Service		2.7 (2.3, 3.3)		0.2 (0.4, 0.6)		36.6 (30.5, 43.1)		0.05 (0.01, 0.3)
None (no coverage)		0.4 (0.2, 0.8)		0.7 (0.3, 1.9)		1.0 (0.3, 3.0)		0.05 (0.01, 0.1)

[a] Weighted percentages using CDC final weights

Table S2. Percentage[a] of women 40 years of age and older by racial/ethnic group by their mammography history, by state for Arizona and New Mexico, BRFSS, 2016 and 2018.

	**All**		**Hispanic**		**AI**		**NHW**	
	**Arizona**<br>**n= 7,806**<br>**% (95% CI)**	**New Mexico**<br>**n= 5,024**<br>**% (95% CI)**	**Arizona**<br>**n= 868**<br>**% (95% CI)**	**New Mexico**<br>**n= 1,499**<br>**% (95% CI)**	**Arizona**<br>**n= 422**<br>**% (95% CI)**	**New Mexico**<br>**n= 432**<br>**% (95% CI)**	**Arizona**<br>**n= 6,516**<br>**% (95% CI)**	**New Mexico**<br>**n= 3,093**<br>**% (95% CI)**
Ever had a mammogram								
Yes	91.6 (90.2, 92.8)	89.7 (88.3, 90.9)	84.7 (79.5, 88.8)	87.4 (84.8, 89.7)	84.6 (78.4, 89.2)	82.4 (76.6, 87.1)	93.7 (92.6, 94.7)	92.4 (90.9, 93.7)
How long since last mammogram								
Within the past year	55.9 (53.9, 57.9)	48.8 (46.7, 50.8)	57.7 (51.7, 63.6)	49.8 (46.2, 53.5)	56.0 (48.1, 63.5)	48.5 (41.6, 55.5)	55.4 (53.4, 57.4)	48.0 (45.4, 50.6)
Within the past two years	19.4 (17.7, 21.1)	22.5 (20.8, 24.3)	21.9 (16.8, 28.0)	25.6 (22.5, 29.1)	20.6 (15.2, 27.4)	24.2 (18.7, 30.7)	18.7 (17.0, 20.4)	20.0 (18.1, 22.2)
Within the past 3 years	8.2 (7.2, 9.3)	10.5 (9.3, 11.8)	6.7 (4.4, 10.2)	9.5 (7.6, 11.8)	8.0 (4.8, 13.2)	14.9 (10.2, 21.3)	8.6 (7.5, 9.8)	10.7 (9.2, 12.4)
Within the past 5 years	6.0 (5.2, 7.0)	7.1 (6.2, 8.1)	7.2 (4.8, 10.6)	6.5 (5.1, 8.4)	4.6 (2.0, 10.2)	6.5 (3.8, 10.8)	5.8 (5.0, 6.7)	7.6 (6.4, 9.0)
Five or more years	10.5 (9.4, 11.7)	11.1 (10.0, 12.4)	6.5 (4.1, 10.0)	8.5 (6.8, 10.6)	10.8 (6.5, 17.5)	5.9 (3.5, 9.6)	11.6 (10.4, 12.9)	13.7 (12.1, 15.5)
U.S. Preventative Services Task Force (USPSTF) Compliant								
40-49, mammogram in past 2 years	57.5 (52.6, 62.3)	49.6 (45.9, 54.9)	56.3 (46.2, 65.9)	50.7 (44.1, 57.3)	57.7 (43.1, 71.0)	32.4 (22.6, 44.1)	58.2 (52.7, 63.6)	52.2 (45.3, 59.0)
50-74, mammogram in past 2 years	74.8 (72.7, 76.8)	72.1 (70.0, 74.1)	75.2 (68.3, 81.0)	74.9 (71.0, 78.3)	68.1 (59.1, 75.8)	74.7 (67.9, 80.5)	74.9 (72.8, 76.9)	69.7 (67.0, 72.3)
75-80+, mammogram in past 2 years	62.8 (59.1, 66.4)	53.3 (48.5, 58.0)	64.7 (50.4, 76.7)	61.5 (52.1, 70.2)	61.2 (32.9, 83.6)	49.9 (29.2, 70.6)	62.7 (58.8, 66.4)	50.0 (44.3, 55.7)
American Cancer Society (ACS) Compliant								
Overall ACS compliance for 40+	62.8 (60.9, 64.7)	57.5 (55.6, 59.5)	59.5 (53.8, 64.9)	57.7 (54.3, 61.0)	55.4 (48.1, 62.5)	51.9 (45.4, 58.3)	64.0 (62.1, 65.9)	58.2, (55.7, 60.6)
40-54, mammogram within past year	45.1 (41.4, 48.9)	39.0 (35.6, 42.6)	42.8 (35.3, 50.6)	37.7 (32.7, 43.0)	35.5 (26.2, 46.0)	27.1 (19.4, 36.5)	46.8 (42.5, 51.0)	43.3 (38.0, 48.7)
55-80+, mammogram in past 2 years	72.9 (71.0, 74.7)	68.1 (66.0, 70.1)	79.5 (73.5, 84.4)	74.2 (70.3, 77.7)	70.8 (61.9, 78.3)	73.3 (65.8, 79.7)	71.7 (69.8, 73.6)	64.0 (61.3, 66.6)
American College of Radiology (ACR) Compliant								
40-80+, mammogram within past year	51.2 (49.2, 53.1)	43.8 (41.8, 45.7)	48.9 (43.3, 54.5)	43.6 (40.2, 47.0)	47.3 (40.3, 54.5)	40.0 (33.9, 46.3)	51.9 (50.0, 53.9)	44.4 (41.9, 46.9)

[a] Weighted percentages using CDC final weights

Table S3. Percentage[a] of women 50-74 years who are USPSTF compliant by sociodemographic/health utilization variables, Arizona and New Mexico, BRFSS, 2016 and 2018.

	All<br>n= 9,984<br>% (95% CI)	Hispanic<br>n= 2,051<br>% (95% CI)	AI<br>n= 793<br>% (95% CI)	NHW<br>n= 7,140<br>% (95% CI)
General health				
Poor to Fair	27.1 (24.9, 29.4)	42.5 (36.8, 48.4)	34.1 (28.0, 40.7)	21.4 (19.3, 23.7)
Good to Excellent	72.9 (70.6, 75.1)	57.5 (51.6, 63.2)	65.9 (59.3, 72.0)	78.6 (76.3, 80.7)
Current medical insurance				
No	4.3 (3.3, 5.6)	11.0 (7.6, 15.8)	4.3 (2.4, 7.4)	2.0 (1.4, 2.9)
Yes	95.7 (94.4, 96.7)	89.0 (84.2, 92.5)	95.8 (92.6, 97.6)	98.0 (97.1, 98.6)
Personal doctor or health care provider				
No	7.3 (6.2, 8.5)	11.0 (8.0, 14.9)	23.8 (18.0, 30.8)	5.1 (4.3, 6.2)
Yes	92.7 (91.5, 93.8)	89.0 (85.1, 92.0)	76.2 (69.2, 82.1)	94.9 (93.8, 95.8)
Visited health care provider in past 12 months				
No	12.9 (11.5, 14.4)	12.6 (9.5, 16.5)	17.8 (12.3, 24.9)	12.7 (11.3, 14.3)
Yes	87.1 (85.7, 88.5)	87.4 (83.5, 90.5)	82.3 (75.1, 87.7)	87.3 (86.7, 88.7)
Cost an issue for not visiting doctor in past 12 months				
No	89.3 (87.8, 90.7)	81.6 (76.7, 85.7)	82.0 (76.4, 86.5)	92.4 (91.0, 93.5)
Yes	10.7 (9.3, 12.2)	18.4 (14.3, 23.3)	18.0 (13.5, 23.6)	7.6 (6.5, 9.0)
Employment status				
Currently not working	26.5 (24.4, 28.7)	37.2 (31.6, 43.2)	36.5 (30.2, 43.3)	22.3 (20.2, 24.4)
Employed for wages or self employed	36.7 (34.6, 38.8)	37.2 (32.0, 42.6)	39.2 (32.6, 46.3)	36.4 (34.1, 38.8)
Retired	36.9 (34.9, 38.9)	25.6 (21.7, 30.1)	24.3 (18.7, 31.0)	41.4 (39.1, 43.7)
Age				
50-54	20.4 (18.5, 22.5)	23.2 (19.0, 27.9)	22.2 (17.0, 28.5)	19.4 (17.2, 21.8)
55-59	20.5 (18.5, 22.7)	28.0 (22.3, 34.6)	23.3 (17.9, 29.8)	17.8 (16.0, 19.7)
60-64	22.4 (20.8, 24.2)	21.2 (17.4, 25.4)	27.8 (21.9, 34.6)	22.6 (20.7, 24.6)
65-69	19.3 (17.4, 20.7)	15.0 (12.1, 18.3)	18.9 (13.9, 25.1)	20.8 (19.1, 22.5)
70-74	17.4 (16.0, 18.9)	12.7 (10.0, 16.0)	7.8 (5.3, 11.3)	19.5 (17.9, 21.2)
Level of education completed				
Did not graduate high school	14.6 (12.5, 16.9)	43.9 (38.1, 49.8)	21.0 (15.5, 27.8)	4.1 (3.1, 5.4)
Graduated high school	21.8 (20.2, 23.5)	20.6 (17.3, 24.4)	30.3 (24.4, 37.0)	21.8 (19.9, 23.8)
Attended college/technical school	37.9 (35.7, 40.0)	24.7 (20.8, 29.1)	35.9 (29.6, 42.7)	42.5 (40.1, 44.9)
Graduated college/technical school	25.8 (24.2, 27.4)	10.9 (8.7, 13.5)	12.8 (9.1, 17.8)	31.6 (29.6, 33.6)
Graduated college/technical school				
No	74.2 (72.6, 75.8)	89.1 (86.5, 91.3)	87.2 (82.3, 90.9)	68.4 (66.4, 70.4)
Yes	25.8 (24.2, 27.4)	10.9 (8.7, 13.5)	12.8 (9.1, 17.8)	31.6 (29.6, 33. 6)
Annual household income				
Less than $15,000	12.6 (10.6, 14.9)	29.0 (22.5, 36.4)	29.6 (23.4, 36.7)	6.1 (5.2, 7.3)
$15,000 to >$25,000	18.3 (16.5, 20.3)	26. 9 (21.9, 32.5)	29.3 (22.9, 36.7)	14.9 (13.1, 16.8)
$25,000 to >$35,000	10.1 (8.9, 11.5)	10.5 (7.8, 14.1)	12.0 (8.2, 17.3)	9.8 (8.5, 11.4)
$35,000 to >$50,000	12.8 (11.5, 14.2)	9.0 (6.7, 11.9)	11.0 (6.7, 17.7)	14.1 (12.6, 15.8)
$50,000 or more	46.3 (43.9, 48.7)	24.7 (20.3, 29.6)	18.0 (13.4, 23.9)	55.1 (52.5, 57.6)
Annual household income $50,000 or more				
No	53.7 (51.3, 56.1)	75.3 (70.4, 79.7)	82.0 (76.1, 86.6)	44.9 (42.4, 47.5)
Yes	46.3 (43.9, 48.7)	24.7 (20.3, 29.6)	18.0 (13.4, 23.9)	55.1 (52.5, 57.6)

[a] Weighted percentages using CDC final weights
